# Retrospective study of the functional and oncological outcomes of conformal sphincter preservation operation in the treatment of very low rectal cancer

**DOI:** 10.1007/s10151-020-02229-2

**Published:** 2020-05-02

**Authors:** G. Sun, Z. Lou, H. Zhang, G. Y. Yu, K. Zheng, X. H. Gao, R. G. Meng, H. F. Gong, E. J. B. Furnée, C. G. Bai, W. Zhang

**Affiliations:** 1 Department of Colorectal Surgery, Changhai Hospital, 168 Changhai Rd, Shanghai, 200433 China; 2 Department of Pathology, Changhai Hospital, Shanghai, China; 3 Department of Surgery, University Medical Center Groningen, Groningen, the Netherlands

**Keywords:** Rectal neoplasms, Surgery, Disease-free survival, Postoperative complications, Margins of excision, Follow-up studies, Neoplasm recurrence, Local, Low anterior resection syndrome

## Abstract

**Background:**

Conformal sphincter preservation operation (CSPO) is a new surgical procedure for very low rectal cancers (within 4–5 cm from the anal verge). CSPO preserves more of the dentate line and distal rectal wall and also avoids injuring nerves in the intersphincteric space, resulting in satisfactory anal function after resection. The aim of this study was to analyze the short-term surgical results and long-term oncological and functional outcomes of CSPO.

**Methods:**

Consecutive patients with very low rectal cancer, who had CSPO between January 2011 and October 2018 at Changhai Hospital, Shanghai were included. Patient demographics, clinicopathological features, oncological outcomes and anal function were analyzed.

**Results:**

A total of 102 patients (67 men) with a mean age of 56.9 ± 10.8 years were included. The median distance of the tumor from the anal verge was 3 (IQR, 3–4) cm. Thirty-five patients received neoadjuvant chemoradiation (nCRT). The median distal resection margin (DRM) was 0.5 (IQR, 0.3–0.8) cm. One patient had a positive DRM. All circumferential margins were negative. There was no perioperative mortality. The postoperative complication rate was 19.6%. The median duration of follow-up was 28 (IQR, 12–45.5) months. The local recurrence rate was 2% and distant metastasis rate was 10.8%. The 3-year overall survival and disease-free survival rates were 100% and 83.9%, respectively. The mean Wexner incontinence and low anterior resection syndrome scores 12 months after ileostomy reversal were 5.9 ± 4.3, and 29.2 ± 6.9, respectively.

**Conclusions:**

For patients with very low rectal cancers, fecal continence can be preserved with CSPO without compromising oncological results.

## Introduction

Very low rectal cancer within 4–5 cm from the anal verge has traditionally been treated by abdominoperineal resection (APR) with acceptable oncological results [1, 2]. However, a permanent stoma decreases patients’ quality of life significantly [3]. With the advancement of surgical oncology and instrumentation, intersphincteric resection (ISR) and coloanal anastomosis (CAA) have gained widespread acceptance. The reduction of the distal resection margin did not seem to impact the incidence of recurrence and long-term survival [4]. However, according to the literature and our experience with ISR, patients often have poor anal function after surgery due to removal of the internal anal sphincter and the dentate line, which are important parts of the anal sphincter complex [5, 6] and also because of the extensive dissection in the intersphincteric space (ISS) which destroys autonomic nerves [7, 8]. The functional problems after ISR lead to a significant decrease in postoperative quality of life [9, 10]. To overcome this functional shortcoming, we designed a new surgical procedure, the conformal sphincter preservation operation (CSPO). CSPO preserves more dentate line and distal rectal wall and also avoids injuring nerves in the intersphincteric space. Besides, the anastomosis is fashioned on the part with more preserved rectal wall, thus the anastomosis ring can be 2–3 cm above the dentate line so as to get more satisfactory anal function after resection. The initial experiences of this procedure have already been published [5]. The aim of the present study was to analyze the short-term surgical results and long-term oncological and functional outcomes of CSPO.

## Materials and methods

### Patient selection

This retrospective study was conducted in the Department of Colorectal Surgery, Changhai Hospital, Shanghai, China. All procedures were performed in accordance with the ethical standards of our institutional research committee and with the 1964 Helsinki Declaration and its later amendments or comparable ethical standards. The demographic, clinicopathological and follow-up information were recorded in our Colorectal Cancer Database. All patients who had CSPO performed by the same surgical team between January 2011 and October 2018 were reviewed. Patients who met all of the following criteria were included into the study: (1) proven rectal adenocarcinoma with digital rectal examination, colonoscopy and biopsy; (2) the tumor did not infiltrate the intersphincteric space; (3) neoadjuvant therapy in the case of preoperative stage T3–T4 or N+ , or if the circumferential margin was considered positive; (4) good anal function before the operation; (5) the distance from the inferior tumor edge to the anal verge was less than 4–5 cm on rectal digital examination; or less than 2 cm from the dentate line on proctoscopy or colonoscopy; (6) the diameter of the tumor was less than 3 cm and occupied less than 1/3 circumference of the lumen (the actual diameter measured after resection may be slightly larger). Exclusion criteria were: (1) distant metastasis including those with metastatic lymph nodes outside of mesorectum; (2) poorly differentiated or undifferentiated cancers; (3) patient unable to tolerate the operation (American Society of Anesthesiologists (ASA) class > 3).

### Surgical technique

**Fig. 1:**
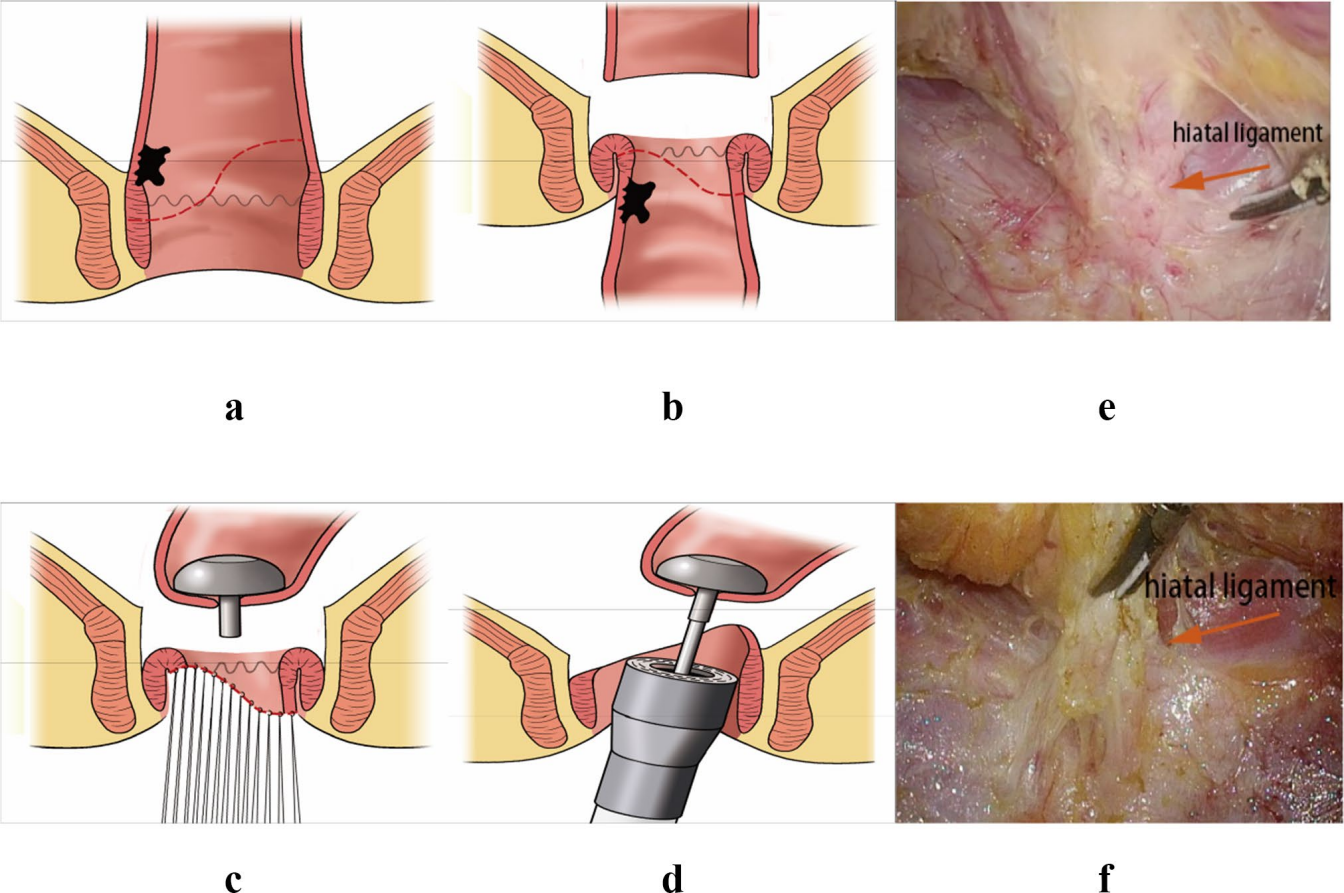
The CSPO technique. **a**, **b** The tumor was pulled out of the anus through the rectal lumen, the distal dissection line was made at least 1 cm below the inferior tumor margin, preserving more rectum on the opposite tumor side. **c** The rectal stump was closed by manual interrupted sutures. **d** The stapler was inserted to the upper tip of the rectal stump to preserve more rectum wall. **e**, **f** The hiatal ligament

**Fig. 2:**
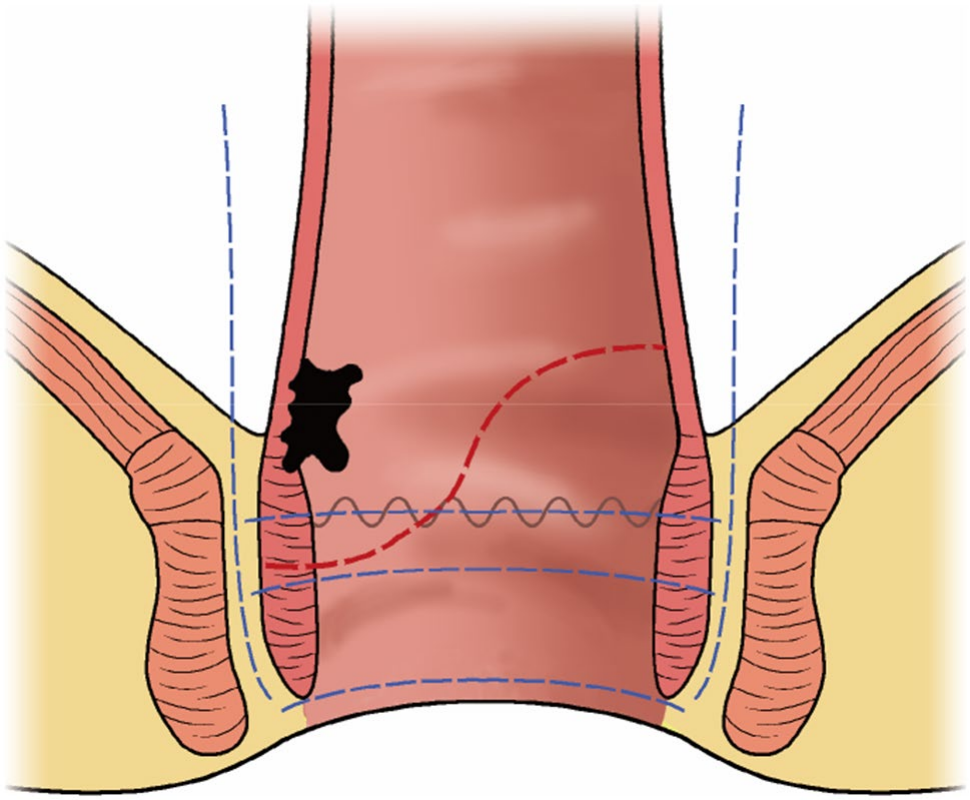
Drawing of the transection lines for ISR (blue lines). Total intersphincteric resection (total-ISR) is defined as an internal sphincter resection at the intersphincteric groove, subtotal-ISR is between the dentate line (DL) and ISG, and partial-ISR is at the DL. But the CSPO stops at the entrance of ISS, and resection line in the internal sphincter is inclined and conformed to the tumor edge

**Fig. 3:**
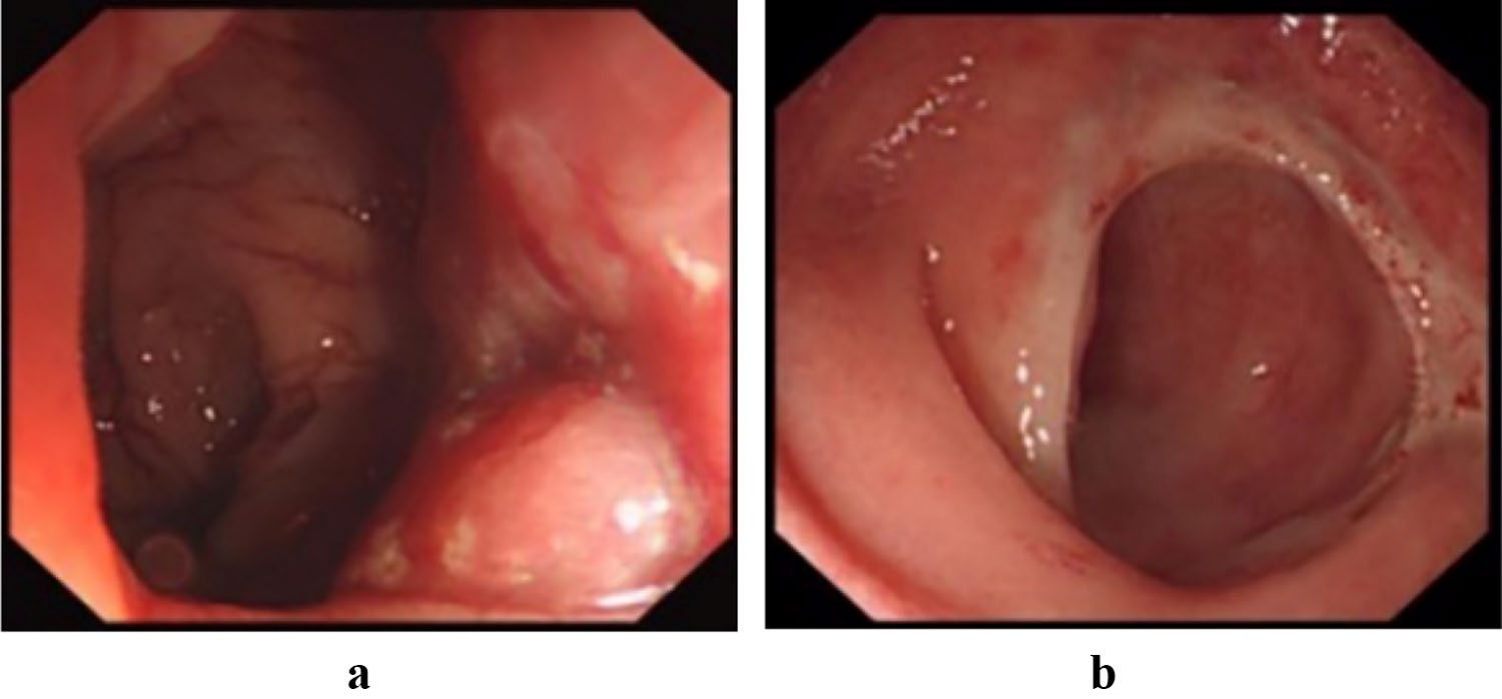
Colonoscopy **a** Preoperative colonoscopy shows that the mass is 1 cm above the dentate line. **b** Five months postoperatively, colonoscopy shows the anastomotic line 2–3 cm away from the dentate line

The CSPO starts with standard mobilization of the sigmoid colon, ligation of the inferior mesenteric artery at its origin and mobilization of the rectum according to the principle of total mesorectal excision (TME) with attention paid to preserve the autonomic nerves. Dissection of the rectum continues downward until reaching the hiatal ligament [11]. After cutting the hiatal ligament (Fig. 1), dissection stops at the entrance of the intersphincteric space, which is one of the main differences between ISR and CSPO (Fig. 2). The rectum is transected at the rectosigmoid junction. Subsequently, the anus is dilated to 3–4 fingers wide and the rectum was pulled out of the anus through the rectal lumen (Fig. 1). The conformal incision line is designed according to the tumor’s location and size. The key point of this procedure is to preserve as much of the lower rectum, dentate line and internal anal sphincter, on the side opposite to the tumor as possible. The intersphincteric space was left undisturbed to prevent injuring the numerous nerve fibres it contains [7] to preserve the function of the remaining internal sphincter. The distal dissection line is made at least 1 cm below the inferior tumor margin under direct vision. In cases where the tumor cannot be inverted from the rectum, in-situ transanal excision can still be performed to preserve adequate rectal wall appropriately. The remnant rectal stump was then closed with interrupted sutures. Intraoperative frozen-section examination was performed in all cases to ensure an adequate oncological DRM. Following the rectal stump closure, a 25 mm circular stapler (CDH25, Johnson & Johnson, USA) was used to perform the anastomosis [12] (Fig. 1). The stapler is inserted as high in the rectal stump as possible, to make the anastomosis as high as possible on the opposite side. Consequently, as much as possible of the dentate line and internal anal sphincter are preserved on the opposite side. Temporary ileostomy was routinely performed in all patients. The anastomotic line is shown in Fig. 3: it is 2–3 cm above the dentate line even though the patient has such a low rectal cancer. Patients with pathological stage III or stage II disease with high-risk features received postoperative Capeox or mFOLFOX6 regimen as adjuvant chemotherapy. Postoperative radiotherapy was performed in patients who had not had preoperative radiotherapy according to the following criteria: (1) pathological result ≥ N1b or circumferential resection margin (CRM) positive; (2) T3 or T4; (3) distal margin too short (usually less than 0.3 cm).

### Ileostomy reversal

The ileostomy was closed 3–6 months after the resection or after finishing adjuvant chemical or chemo-radio therapy, depending on the clinical fitness of the patient and the following criteria: (1) the digital rectal examination was performed every 4 weeks after the resection to make sure the anastomotic ring was smooth, complete and no stenosis; (2) local recurrence excluded by pelvic magnetic resonance imaging (MRI), a chest computed tomography (CT) scan and MRI of liver excluded distant metastases; (3) defecography with iodized water was performed when there was any suspicion of an anastomotic leakage; (4) colonoscopy was performed to make sure that there was no stenosis and local recurrence at the anastomosis and that the anastomotic line was complete and with no obstruction in the proximal bowel.

### Oncological and functional follow-up

Patients were followed every 3 months for the first 2 years, every 6 months for the next 3 years, and once a year thereafter. Digital rectal examination, carcinoembryonic antigen and carbohydrate antigen 19-9 test were performed at every follow-up visit; chest CT scan, MRI of the liver and pelvis with intravenous contrast and colonoscopy was performed according to the National Comprehensive Cancer Network (NCCN) guidelines. Postoperative complications were graded according to the Clavien–Dindo classification system [13]. Anal function was assessed 12 months after ileostomy closure using the Wexner fecal incontinence and low anterior resection syndrome (LARS) score [14, 15].

### Statistical analysis

The continuous variables were reported as mean ± standard deviation (SD) or as median with interquartile ranges (IQR), depending on whether the data were normally distributed or not. Categorical variables were statistically analyzed by the Chi-square test and continuous variables were compared using Student’s *t* test or Mann–Whitney test. The baseline characteristics and follow-up results were compared between the patients with and without neoadjuvant chemoradiotherapy (nCRT). The Kaplan–Meier method and log rank test were used for analysis of prognostic factors for disease-free survival, overall survival and stoma-free survival. A *p* value < 0.05 (two sided) was considered statistically significant. Statistical analysis was performed using IBM SPSS Statistics 23.0 (IBM SPSS Statistics, IBM Corporation, Armonk, NY, USA). Figures were generated using GraphPad Prism 7.02 (GraphPad Software Inc., La Jolla, CA, USA).

## Results

### Demographic and clinicopathological characteristics

A total of 102 patients (mean age 56.9 ± 10.8 years) met the inclusion criteria and had CSPO between January 2011 and October 2018. The mean body mass index was 23.0 ± 3.3. A total of 35 patients (34.3%) received nCRT followed by resection 6–8 weeks after the completion of the neoadjuvant therapy. The other 67 patients (65.7%) were treated by surgery without neoadjuvant treatment. Seventeen patients (16.7%) received postoperative radiotherapy. The approach was by laparoscopy in 38 patients (37.3%), including 1 (2.6%) conversions to open surgery, and 64 (62.7%) were open procedures. The median tumor distance from the anal verge was 3 (IQR, 3–4) cm.

The colorectal (or coloanal) anastomoses were performed with stapling except in one case when the tumor was too low for a stapled anastomosis and a hand-sewn anastomosis was performed. One planned CSPO was converted to APR, because two consecutive intraoperative frozen-section examinations showed tumor cells at the resection margin, despite absence of visible tumor. The median DRM was 0.5 (IQR, 0.3–0.8) cm and all patients had R0 resection and negative CRM, except one patient who had a positive DRM (< 1 mm). The median number of lymph nodes retrieved was 14 (IQR, 10–15.25) and the median tumor diameter was 2.7 (IQR, 2–3.5) cm. Complete pathological response to neoadjuvant therapy was achieved in 7 of the 35 patients who received nCRT (20.0%). Comparing the nCRT and non-nCRT group, age was significantly lower, the tumor’s largest diameter was significantly smaller and cT stage and cN stage were more advanced in the nCRT group (Table 1).

**Table 1:** Baseline characteristics

	Total (*n* = 102)	nCRT (*n* = 35)	Non-nCRT (*n* = 67)	p*
Age, (years)	56.9 ± 10.8	53.7 ± 9.4	58.5 ± 11.2	*0.03*
Male sex	67 (65.7%)	20 (57.1%)	47 (70.1%)	0.19
Body mass index (kg/m^2^)	23.0 ± 3.3	22.3 ± 3.1	23.4 ± 3.3	0.12
Tumor location (cm)^a^	3 (3–4)	3 (3–4)	3 (3–4)	0.84
Estimated blood loss (ml)	143.3 ± 101.7	147.4 ± 78.0	141.1 ± 112.6	0.77
Laparoscopic surgery	38 (37.3%)	11 (31.4%)	27 (40.3%)	0.38
Operative time (min)	166.9 ± 55.2	171.9 ± 56.5	164.2 ± 54.7	0.51
Postoperative hospital stay (days)	6.8 ± 2.7	6.5 ± 2.3	6.9 ± 2.9	0.41
Distal resection margins (cm)	0.5 (0.3–0.8)	0.5 (0.3–1.0)	0.5 (0.3–0.8)	0.52
Lymph nodes retrieval number	14 (10–15)	10 (5–14)	15 (13–16)	< *0.001*
Tumor diameter (cm)	2.7 (2–3.5)	2 (1.5–2.5)	3 (2.5–4)	< *0.001*
cT stage				< *0.001*
T1	16 (15.7%)	0 (0%)	16 (23.9%)	
T2	53 (52.0%)	17 (48.6%)	36 (53.7%)	
T3	32 (31.4%)	17 (48.6%)	15 (22.4%)	
T4	1 (1%)	1 (2.9%)	0 (0%)	
cN stage				0.001
N0	55 (53.9%)	11 (31.4%)	44 (65.7%)	
N1–2	47 (46.1%)	24 (68.6%)	23 (34.3%)	
pT stage				0.51
T0	7 (6.9%)	7 (20%)	0 (0%)	
T1	21 (20.6%)	3 (8.6%)	18 (26.9%)	
T2	53 (52.0%)	18 (51.4%)	35 (52.2%)	
T3	21 (20.6%)	7 (20%)	14 (20.9%)	
pN stage				0.52
N0	84 (82.4%)	30 (85.7%)	54 (80.6%)	
N1–2	18 (17.6%)	5 (14.3%)	13 (19.4%)	
Pathological stage (TNM)^b^				0.51
0	7 (6.9%)	7 (20%)	0	
I	65 (63.7%)	20 (57.1%)	45 (67.2%)	
II	14 (13.7%)	4 (11.4%)	10 (14.9%)	
III	16 (15.7%)	4 (11.4%)	12 (17.9%)	

*nCRT* neoadjuvant chemoradiotherapy

*Difference between nCRT and non-nCRT group

**Values are reported as mean ± SD or as median and interquartile range

^a^Distal edge of the tumor to the anal verge

^b^TNM stage according to AJCC 8th edition

### Complications

There was no 30-day mortality. The overall complication rate was 19.6%. According to the Clavien–Dindo classification [13], overall incidence of grade 2 or higher postoperative complications was 14.7% (15/102). The details of complications in both groups are reported in Table 2.

**Table 2:** Complications after CSPO

Clavien–Dindo Grade	Complication	Total (*n* = 102)	nCRT (*n* = 35)	Non-nCRT (*n* = 67)
Grade ≥ I, *n *(%)		20 (19.6)	8 (22.9)	12 (17.9)
Grade I, *n*	Wound infection	2	0	2
	Bladder retention	1	0	1
	Incisional hernia	1	0	1
	Inguinal hernia	1	1	0
Grade II, *n*	Urinary infection	1	0	1
	Anastomotic leakage	1	0	1
	Intestinal obstruction	5	2	3
	Radiation proctitis	1	1	0
	Rectovaginal fistula	1	0	1
	Anastomotic stricture	3	3	0
	Pneumonia	1	0	1
Grade IIIa, *n*	Anastomotic leakage	1	1	0
Grade IIIb, *n*	Intestinal obstruction	1	0	1

*nCRT* neoadjuvant chemoradiotherapy, *CSPO* conformal sphincter preservation operation

### Oncological results

**Fig. 4:**
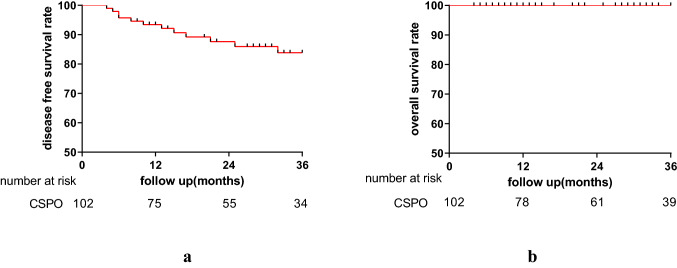
Survival after CSPO. **a** Disease-free survival, **b** overall survival

The median duration of follow-up was 28 (IQR, 12–35) months. The disease-free survival rates at 1, 2 and 3 years were 92.5%, 86.3% and 83%, respectively (Fig. 4). The 3-year local recurrence-free and distant recurrence-free survival rates were 97.5% and 86%, respectively. The local recurrence rate was 2% and the distant metastasis rate was 10.8%.

### Ileostomy reversal

**Fig. 5:**
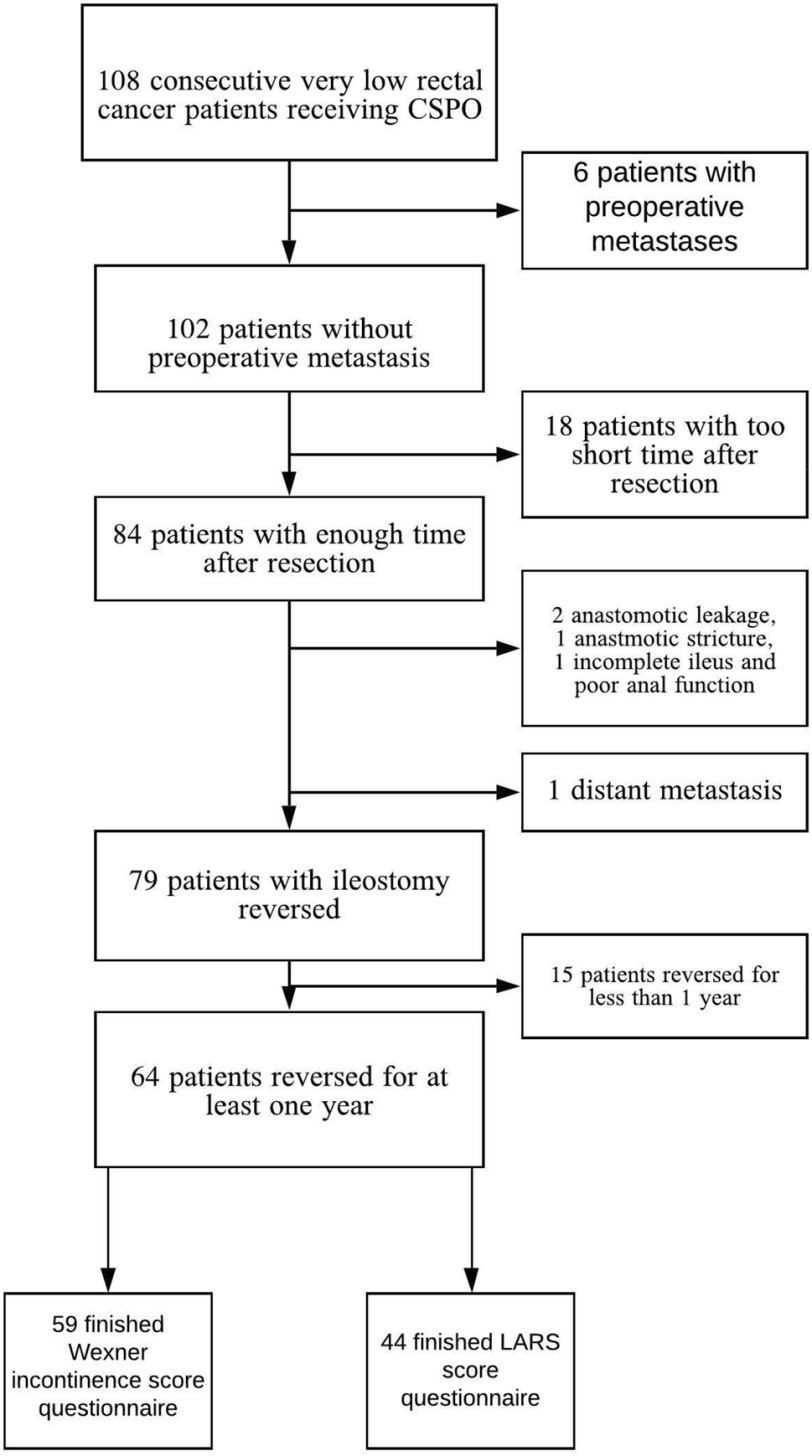
Flow chart of patients

**Fig. 6:**
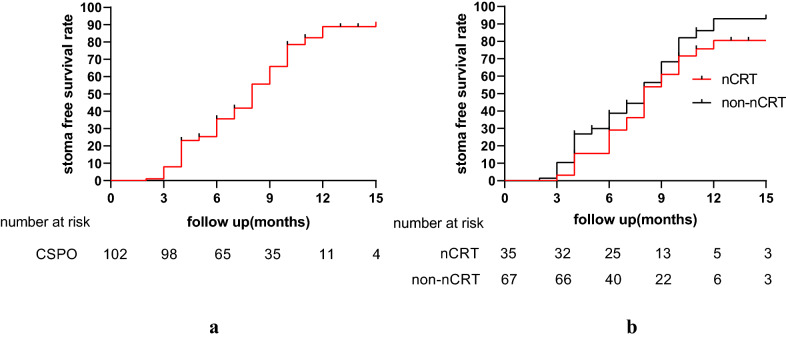
Stoma free survival of patients after CSPO. **a** Stoma free survival of all the patients after CSPO. **b** Stoma free survival for patients with and without nCRT, *p* = 0.23

The ileostomy was reversed in 79 patients (77.5%; Fig. 5) with a median time to reversal of 7 (IQR, 4–10) months. The rates of ileostomy reversal in the nCRT and non-nCRT groups was 71.4% (25/35) and 80.6% (54/67), respectively, however, the difference was not statistically significant (*p* = 0.29). The median time to ileostomy reversal for nCRT and non-nCRT patients was 8 (IQR, 5.0–9.5) and 7 (IQR, 4–10) months, respectively. However, the difference was not statistically significant (*p* = 0.72). The ileostomy-free survival is shown in Fig. 6.

### Functional results

The mean Wexner incontinence and LARS score were 5.9 ± 4.3, and 29.2 ± 6.9. A total of five patients (11.4%) had no LARS (score 0–20), 18 (40.9%) minor LARS (score 21–29), 21 (47.7%) major LARS (score 30–42). The mean Wexner score with or without nCRT was 7.1 ± 5.0 and 5.3 ± 4.0, respectively (*p* = 0.15). The mean LARS score with or without nCRT was 31.4 ± 6.4 and 28.4 ± 6.9 (*p* = 0.19). For the postoperative radiotherapy patients, the mean Wexner score was 7.3 ± 4.1 (*p* = 0.92, in comparison to nCRT patients), the mean LARS score was 29.6 ± 1.9 (*p* = 0.47, in comparison to nCRT patients), 12 months after ileostomy reversal.

## Discussion

The results of our study suggest that CSPO is safe with acceptable oncological and functional outcomes and can be performed in selected patients with very low, small and early-stage rectal cancer. CSPO, therefore, has a role as a sphincter saving procedure, with the advantages of achieving a balance between oncologic safety and functional results.

With the development of surgical techniques such as the pull-through technique, ISR and low anterior resection (LAR) with the double stapling method, the rate of sphincter preservation in operations for rectal cancer has improved [16]. ISR, an anus-preserving technique with internal anal sphincter muscle resection, described by Schiessel in 1994 [17], has become popular around the world [8, 18]. ISR for T1–3 tumors located within 30–35 mm from the anal verge is technically feasible, safe, and with equal oncological outcomes compared to conventional surgery [19]. However, Wexner fecal incontinence scores after ISR were significantly higher compared with LAR, thus negatively influencing the postoperative quality of life [20]. According to Johannes et al. [21], 80% of the 60 patients’ pathological TNM stages were lower than III in ISR, similar to the value of 84% in CSPO, while, the mean Wexner score was reported to be 10.6 after ISR, higher than 5.9 after CSPO in the current study. According to Ahmad Sakr et al. [22], 70.8% of patients had pathological TNM stages 0 or I after ultralow anterior resection, similar to the 70.6% of patients treated with CSPO, while the median Wexner incontinence score was 10 after ultralow anterior resection, which was higher than 5.9 after CSPO seen in our series. This may be because more rectal wall, dentate line and anal canal was preserved by the conformal resection [23]. For some tumors 3–4 cm from the anal verge, the anastomotic line can still be 2–3 cm above the dentate line in CSPO, which helps preserving the anal function. In addition, the nerves in the intersphincteric space were left undisturbed as we avoid dissection in the intersphincteric space during CSPO.

Kim et al. [24] reported the minimum distance of tumor from the anal verge before APR is unavoidable is 3.4 cm, compared to 3 cm in the current CSPO group (however, with more advanced cancers in the former group). Based on the distance from the inferior tumor edge to the anal verge, CSPO allowed sphincter preservation in patients who would otherwise have had APR, and with acceptable anal function after surgery.

The median DRM was just 0.5 cm in the pathologic specimens in the current study, but the clinical DRM during CSPO was about 1 cm to ensure oncological safety and accurate resection under direct vision. This is consistent with the literature [25, 26]. Because of the bowel shrinkage occurring during the first 10–20 min after removal from the patients and additional shrinkage after formalin fixation, a correction factor of 50% reduction in anatomically non-restored fixed specimens has been proposed [25, 26]. However, a recent systematic review found no negative impact on oncological safety when the pathological fixed DRM was < 5 mm in a group of patients with favorable tumors [27–29]. Even though 78% of DRMs were less than 1 cm in the pathological examination in the current study, the positive DRM margin rate was only 0.98% (one patient). In addition to the effect of neoadjuvant chemoradiotherapy in 35 patients, this probably results from the accurate dissection under direct vision during the pull-through part of the CSPO procedure, avoiding the difficulty of palpation and locating the tumor margin in a narrow pelvis during the abdominal approach, especially when encountering the tumor shrinkage after neoadjuvant therapy.

Three-year disease-free survival and overall survival after CSPO were 83.9% and 100%, respectively. Importantly, local recurrence and distant metastasis rates were 2% and 10.8%, respectively, at a median follow-up of 28 months. Actually, the local recurrence rate after rectal sphincter preserving surgery combined with radiotherapy has been reported to be 4–17% and might be higher after surgery without neoadjuvant therapy [30–34]. The reason for these lower numbers in the current study might be due to lower T and N stage patients in the CSPO group. The complete pathological response rate of 20% in nCRT patients is in accordance with the complete pathological response rate of 14–20% reported in the literature [35–38]. The tumor diameter was also relatively smaller after nCRT, which could facilitate the pull-through procedure, making the resection easier as well as decreasing the squeezing of the tumor during the pull-through procedure thus lowering the risk of spill of tumor cells and hereby the chance of developing a local recurrence.

In the current study, we chose to use the 25 mm circular stapler rather than a larger size circular stapler to avoid trauma to the anal canal [39, 40]. Importantly, rectal digital examination was performed every month during follow-up to prevent anastomotic strictures. The postoperative anastomotic stricture rate was acceptable with this smaller stapler: the incidence of anastomotic stricture in CSPO was 3.9% (three membranous and one fibrotic stricture), lower than the corresponding figures of 7.8% in ISR reported by Soo Young Lee et al. [41].

Radiotherapy can damage anal function. The Wexner incontinence score, LARS score and stoma-free survival rate were found to be better in patients without neoadjuvant radiotherapy in the current study. However, it was very interesting to find that there was no significant difference, as radiotherapy was found to be a risk factor for low anterior resection syndrome [42], influence fecal continence [43] and delay ileostomy reversal [44]. This was not a finding in our study, perhaps because we had a study population with early rectal cancer and therefore a limited part of them received neoadjuvant chemoradiation.

This study has limitations. One limitation is the retrospective study design without a control group to compare with CSPO. Since we have stopped performing ISR in our department due to the disturbed anal function after this operation, we do not have a suitable comparison group. Another limitation is that the cT and cN stage was different between patients with and without nCRT. This also shows that CSPO can be a good choice for some early-stage patients. For some more advanced tumors, CSPO can also be an alternative choice to preserve the anus, but these patients require nCRT.

## Conclusions

CSPO provides an alternative sphincter preserving procedure to treat very low rectal cancer, which is too low to be treated by LAR and until now required APR. In addition, CSPO using appropriate selection criteria is associated with acceptable oncological and functional outcomes, especially in patients with a smaller tumor diameter and earlier stage rectal cancer. Further comparison with ISR is needed.

## Data Availability

The datasets during and/or analyzed during the current study are available from the corresponding author on reasonable request.
